# On the Development of Speech Resources for the Mixtec Language

**DOI:** 10.1155/2013/170649

**Published:** 2013-04-16

**Authors:** Santiago-Omar Caballero-Morales

**Affiliations:** Technological University of the Mixteca, Road to Acatlima K.m. 2.5, 69000 Huajuapan de León, OAX, Mexico

## Abstract

The Mixtec language is one of the main native languages in Mexico. In general, due to urbanization, discrimination, and limited attempts to promote the culture, the native languages are disappearing. Most of the information available about the Mixtec language is in written form as in dictionaries which, although including examples about how to pronounce the Mixtec words, are not as reliable as listening to the correct pronunciation from a native speaker. Formal acoustic resources, as speech corpora, are almost non-existent for the Mixtec, and no speech technologies are known to have been developed for it. This paper presents the development of the following resources for the Mixtec language: (1) a speech database of traditional narratives of the Mixtec culture spoken by a native speaker (labelled at the phonetic and orthographic levels by means of spectral analysis) and (2) a native speaker-adaptive automatic speech recognition (ASR) system (trained with the speech database) integrated with a Mixtec-to-Spanish/Spanish-to-Mixtec text translator. The speech database, although small and limited to a single variant, was reliable enough to build the multiuser speech application which presented a mean recognition/translation performance up to 94.36% in experiments with non-native speakers (the target users).

## 1. Introduction

Research on spoken language technology has led to the development of automatic speech recognition (ASR), Text-to-Speech (TTS) synthesis, and dialogue systems. These systems are now used for different applications such as in mobile telephones for voice dialing, GPS navigation, information retrieval, dictation [1–3], translation [4, 5], and assistance for handicapped people [6, 7].

ASR technology has been used also for language learning, and examples of these can be found in [8–10] for English, [11] for Spanish and French among others, and [12] for “sign” languages. These interfaces allow the user to practice their pronunciation at home or work without the limitations of a schedule. These also have the advantage of mobility as some of them can be installed in different computer platforms or even mobile telephones for basic practicing. However, although there are applications for the most common foreign languages, there are limited (if any) applications for native or ancient languages.

**Figure 1:**
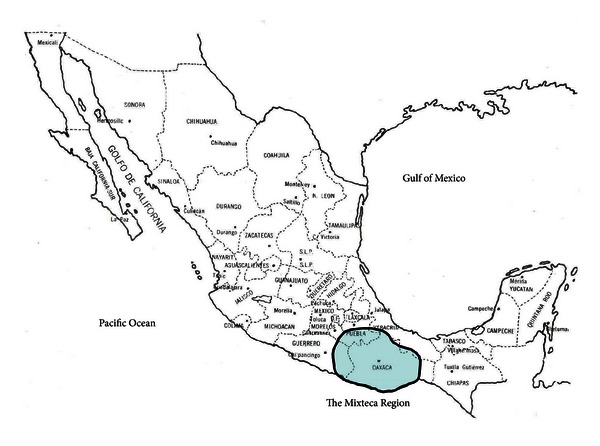
Mixteca Region within the Mexican territory.

In Mexico there are around 89 native languages still spoken by 6.6 millions of native speakers. Although the number of speakers may be significant (considering the total number of inhabitants in Mexico), this number is decreasing, specially in the south-central region, which includes a region known as “La Mixteca” (The Mixteca Region). This region covers parts of the states of Puebla, Guerrero, and Oaxaca as shown in Figure 1. The native inhabitants of The Mixteca are known as “The Mixtec”, and their origin can be traced back to 1500 B.C. As with other native groups in Mexico, their presence has been in decline since the beginning of the Spanish colonization of the Americas.

Nowadays, the population of native speakers of the Mixtec language is further decreasing given urban migration and development, culture rejection, and limited attempts to preserve the language. This has been expressed by people living in communities in The Mixteca Region of Mexico, and this can be corroborated by national statistics that show that the number of people who spoke any native language, 6.3 millions in 2000 (7.1% of the total population), decreased to 6.0 millions in 2005 (6.6% of the total population), and this amount was even higher in 1990 (7.5% of the total population) [13]. This increases the possibility of native languages being lost, as some dialects or variations had less than 10 known speakers (i.e., Ayapaneco, 4 speakers; Chinanteco of Sochiapan, 2 speakers; Mixtec of the Mazateca Region, 6 speakers [13]). In this case, historic antecedents or information about the language is not recorded, making it very difficult to recover or save some parts of the language. This may happen to other languages with more speakers. The Mixtec language, with approximately 480,000 speakers, has been reported to lose annually 200 speakers.

To preserve a language is not an easy task, because all characteristics such as grammar rules, written expression, speech articulation, and phonetics must be documented and recorded. Although there are books and dictionaries that among the word definitions include examples about how to pronounce them, this is not as complete as listening to the correct pronunciation from a native speaker. This goal is considered to be attainable by the use of modern technology such as that used for foreign language learning [10, 11] to promote the language among non-native speakers and, thus, to contribute to its preservation.

However, for the Mixtec, any of the goals of preservation, learning/teaching, or promotion of the language is very limited as the availability of native speech corpora is almost non-existent for the development of any speech application. There are many challenges due to the wide range of variations of the Mixtec language, the limited availability of native speakers willing to participate in such projects (e.g., creating training speech corpora), and their lack of knowledge about the formal writing and phonology of the language. Because of this situation, the development of a Mixtec ASR system with large vocabulary is not achievable. Thus, the use of a speaker adaptation technique on a one-native speaker ASR system was studied.

Hence, this paper presents the development of two major resources: (1) a single native speech corpus of a variation of the Mixtec language, labelled at the phonetic and orthographic levels, and (2) two speech applications: (a) an ASR system and (b) a Mixtec-to-Spanish/Spanish-to-Mixtec speech translator. Non-native speakers were considered the target users for these applications because it is important to arouse the interest in the language of the population with more presence in Mexico, the Spanish-speaking people. This may contribute to a change in attitude towards not only the language, but also towards the culture itself, which can be more beneficial for the purposes of preservation.

The speech applications, trained with the native speech database, performed with recognition accuracies up to 94% when tested by non-native speakers, providing meaningful results about the reliability of the database for the development of basic ASR systems. At this point it is important to mention that the ASR systems recognize Mixtec vocabulary by means of speaker adaptation and that assessment of Mixtec pronunciation by the non-native speakers is not performed. Thus, the ASR systems should not be used for the task of evaluation of the validity of a non-native speaker's pronunciation. However the Mixtec speech corpus can be used to perform the research needed for that purpose.

The structure of the paper is as follows: in Section 2 the general characteristics of the Mixtec language variation used for this work are presented, while in Section 3 the building process of the native speech database with this variation are presented. Then, in Section 4 the design of the speech application systems, which includes the supervised training of the system's acoustic models, and the adaptation technique for its use by non-native users, are presented. The details of the testing methodology by the non-native users and the performance of the developed systems are then presented and analyzed in Section 5. Finally, in Section 6 the conclusions and future work are discussed.

## 2. The Mixtec Language

The Mixtec language, or “Tu'un Savi” (Tongue/Language of the Rain) [14], is present mainly in the states of Guerrero, Puebla, and Oaxaca. However, some variations of the language are also present in the states of Sinaloa, Jalisco, and Yucatán. With a number of speakers of approximately 480,000, this is one of the main native or indigenous languages in Mexico. The Mixtec is a tonal language [14], where the meaning of a word relies on its tone, and because of the geographic dispersion of the Mixtec population, there are differences in tones, pronunciations, and vocabularies between communities, which in some cases restricts the communication between them [15]. Because of this, each variation of the Mixtec language is identified by the name of a community, for example, Mixtec from Tezoatlán [16], Mixtec from Yosondúa [17], or Mixtec of Xochapa [18]; existing significant differences between vocabularies and their meanings: “cat” and “mouse” are, respectively, referenced as “chító” and “tiín” by the Mixtec of Silacayoapan and as “vilo” and “choto” by the Mixtec of the South East of Nochixtlán. Hence, the Mixtec cannot be considered as a single and homogeneous language, and there is still a debate about its number of variations. The National Indigenous Languages Institute (better known by its acronym INALI) in Mexico has identified 81 variations [19], while the Summer Institute of Linguistics has identified 30 variations [20].

Because of this diversity there is no conscensus about a standardized phonetic alphabet for the Mixtec. Thus, continuous revision of the Mixtec alphabet is performed by native and non-native researchers of the language. The Academy of the Mixtec Language “Ve'e Tu'un Savi” (House of the Language of the Rain) [14] identified eight vowels and 20 consonants, pointing out that in some variants only five, six, or seven vowels are used. In contrast, the Summer Institute of Linguistics (SIL) [18] identified five vowels and 22 consonants for the Mixtec of Xochapa in Guerrero. In addition to these differences in phoneme definitions, the tones are also subject to uncertainty. Although generally only three tones are identified (high, medium, and low), other researchers have identified up to 20 tones [14]. Hence, even for the native community of researchers, continuous revisions of the phoneme alphabet are performed.

Because of this, a reduced version of the different Mixtec phoneme alphabets was established for the labelling of the speech corpus and the construction of the ASR applications. This is explained in the following section.

### 2.1. Phonetics

In general, the Mixtec has three characteristic tones: high, medium, and low [14, 16–18, 21–24]. In Table 1 some examples of words that change their meanings based on the tone applied to their vowels are shown, where (_) is used to identify the low tone, (′) the high tone, and the medium tone is left unmarked [14]. Although there are other tone representations, where the low tone also is represented with a horizontal line over the vowel [21], usually the high tone is represented with the diacritical (′). The tones are applied on the vowels, and for this work, the standard five vowels /a/, /e/, /i/, /o/, and /u/ were selected.

Based on the phonemes identified in [14, 18, 21–24] and by integrating the different tones in the vowels, the repertoire shown in Table 2 was defined. The Mixtec phonemes are represented in terms of the International Phonetic Alphabet (IPA) and the Spanish Mexican Phonetic Alphabet (Mexbet) [25]. For the Mixtec vowels, the low tone is represented by the diacritical ($\overset{`}{\;}$) while the high tone is represented by ($\overset{´}{\;}$), and the medium tone is unmarked to keep consistency.

The phonetics of the Mixtec has some differences when compared with the Mexican Spanish language. For example, from Table 2:

- the Mixtec phoneme /dj/ represents a phoneme equivalent to the Mexican Spanish phoneme /s/ [26], while /nd/ represents the composition of the sequence /n/+/d/ as in the Mixtec word “ndí” (light); the same applies to /ng/ which represents the sequence of /n/+/g/ as in the word “súngòo” (to settle);
- the Mixtec phoneme /sh/ is pronounced as /∫/ in the English word “she”; in contrast, /ch/ is pronounced as /$\overset{⌢}{ʧ}$/ in the word “change”;
- there are short pauses, uttered as a glottal closure between vowels within a word, which are represented by /'/ such as in the words “tu'un” (language) or “ndá'a” (hand); however, in other words such as “ka'avi” (to study) and “ndá'aita” (bouquet), /'/ does not represent a short pause; instead it represents the extension of the vowel. Hence, phonetic labelling of Mixtec speech requires special attention. In the IPA, the pause is represented by the glottal stop /*ʔ*/;
- the Mixtec phoneme /n/ is pronounced as in the Mexican Spanish word “Nada” (or as in the English word “Nothing”) if it is placed before a vowel, but is mute if placed after the vowel.

For the Mexican Spanish, the inclusion of the archiphonemes /_D/, /_G/, /_N/, and /_R/ in Mexbet was proposed to define the neutralization of the following couples of phonemes: /d/-/t/, /g/-/k/, /n/-/m/, and /*ɾ*/-/r/ [25]. To represent the pronunciation of the sequence of phonemes /k/ and /s/ (as in the English word “extra”), the phoneme /ks/ was added. For both alphabets (Mixtec and Mexican Spanish), the /sil/ phoneme was added to represent the silence.

## 3. The Mixtec Speech Corpus

**Figure 2:**
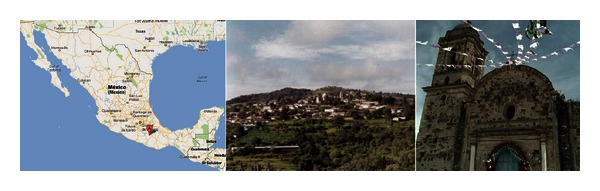
San Juan Diquiyú, place of origin of the Mixtec variant for the speech corpus.

The vocabulary and representative text for the speech corpus were taken from educational material designed by Professor Maximino Sánchez Ventura from the local Cultural Center of the city of Huajuapan de León (Oaxaca, México). He is a native speaker of the Mixtec variant of San Juan Diquiyú (see Figure 2) which is located at the south of Huajuapan de León, in the municipality of Tezoatlán de Segura y Luna. San Juan Diquiyú has a population of approximately 556. Because the Mixtec of San Juan Duquiyú shares similarities with other variations in Oaxaca, there was confidence about using it as the reference variation.

**Figure 3:**
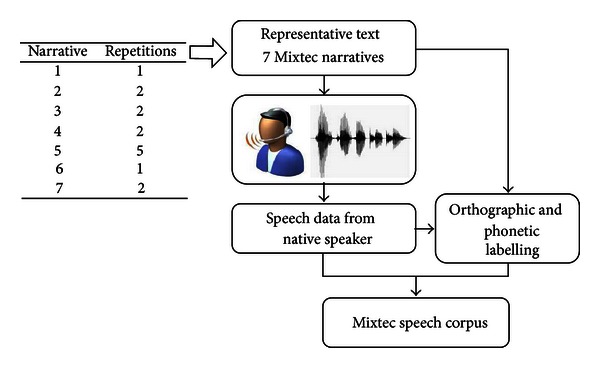
Steps to obtain the Mixtec speech corpus.

**Figure 4:**
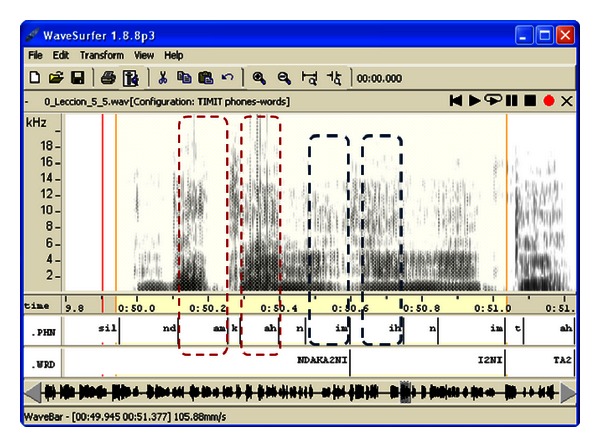
Orthographic and phonetic labelling of the Mixtec speech corpus.

The educational material of Professor Maximino consisted of a collection of 15 traditional Mixtec narratives. For this work, seven were selected, where the first narratives were used for beginners and the last ones for more advanced non-native learners. In Figure 3 the process followed to obtain the speech corpus from the Mixtec narratives is shown. Each narrative was read a certain number of times (see Figure 3) by Professor Maximino, the reference native speaker. These repetitions were recorded in the Media Lab of the Technological University of the Mixteca in WAV format with a sampling rate of 44,100 Hz and one audio channel (monaural). Approximately 45 minutes of native Mixtec speech was recorded. These recordings were then transcribed at the phonetic and word levels (TIMIT standard) using the list of phonemes defined in Table 2, the assistance of Professor Maximino, and spectral analysis using the software WaveSurfer as presented in Figure 4. Note the spectral differences given by the tones between the vowels /a/ (am = /a/ medium tone) and /á/ (ah = /a/ high tone) and /i/ (im) and /í/ (ih).

**Figure 5:**
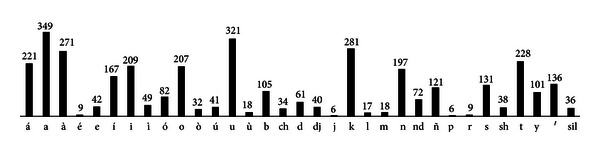
Frequency distribution of phonemes in the Mixtec speech corpus.

In total, the Mixtec speech corpus consisted of 931 words with a vocabulary of 192 (unique) words. The frequency (number of occurrences) of the vocabulary words is presented in Table 3. These words have the frequency of phonemes presented in Figure 5. Based on the phoneme distributions of Figure 5, it was considered that the corpus contained enough samples from each Mixtec phoneme for the supervised training of the acoustic models of an ASR system. A minimum of six was established given the experiments reported in [27] where, for disordered speech, an ASR system was able to achieve accuracies up to 100% when trained with at least six samples of a word. It is important to mention that the phonemes /g/ and /ng/ are not present in this variant; thus, these were not considered. 

## 4. Mixtec Speech Applications

**Figure 6:**
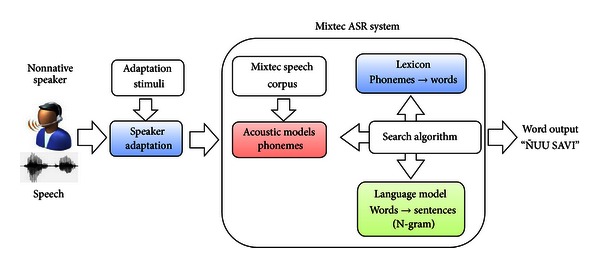
Elements of the speaker-adaptive Mixtec ASR system.

The initial application built with the Mixtec speech corpus was a speaker-adaptive ASR system. The main elements of this system, which became the baseline for more complex applications, are shown in Figure 6. In the following sections a description of the construction of each of these elements is presented.

### 4.1. Speaker-Adaptive ASR Baseline System

An ASR system uses a Bayesian approach to estimate the most likely word sequence $\hat{W}$ out of all possible legal word sentences from a language model *L* given some acoustic input *O*:

(1) $$\begin{array}{c} \hat{W}=\underset{W\in L}{\arg\;\max}\;\text{Pr}\left(W\;|\;O\right). \end{array}$$

The language model contains all the possible word output sequences for a certain application, and the acoustic input is the speech signal. By using Bayes' rule, (1) can be expressed as follows:

(2) $$\begin{array}{c} \hat{W}=\underset{W\in L}{\arg\;\max}\frac{\text{Pr}\left(O\;|\;W\right)\text{Pr}\left(W\right)}{\text{Pr}\left(O\right)}. \end{array}$$

Although (Pr(*O* | *W*)Pr(*W*))/(Pr(*O*)) is estimated for each possible sentence in the language, Pr(*O*) does not change for each sentence and thus can be ignored in (2). Hence,

(3) $$\begin{array}{c} \hat{W}=\underset{W\in L}{\arg\;\max}\;\text{Pr}\left(O\;|\;W\right)\text{Pr}\left(W\right). \end{array}$$

Hence, the most likely sequence of words $\hat{W}$ given some acoustic observation *O* can be estimated as the product of two probabilities [28]:

- Pr(*W*), the *prior probability*, which is obtained from the *language model L*;
- Pr(*O* | *W*), the *observation likelihood*, which is obtained from the *acoustic model*.

Pr(*W*) is usually estimated/modelled by using *N*-gram grammars, and Pr(*O* | *W*) by using Hidden Markov Models (HMMs) [29] or artificial neural networks (ANN).

In this work the modules of the software HTK [30] were used to build the elements of the Mixtec baseline ASR system which include the *N*-grams and the acoustic models. The details are presented in the following sections.

#### 4.1.1. Acoustic Models (Pr(*O* | *W*))

**Figure 7:**
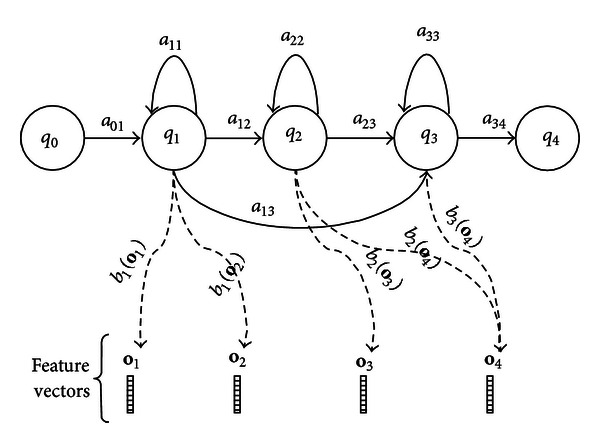
Three-state left-to-right topology of a phoneme HMM.

Hidden Markov Models (HMMs) [28–30] were used for the acoustic modelling of each phoneme in the Mixtec speech corpus. In general terms, an HMM consists of the following parameters which are presented in Figure 7:

(i) a set of states *Q* = {*q*_0_, *q*_1_,…, *q*_*N*_}, where *q*_0_ and *q*_*N*_ are non-emitting states (not associated with observations). Each state has associated a probability function which models the emission of certain acoustic observations (see *B* = {*b*_*j*_(**o**_*t*_)} later);
(ii) a transition probability matrix *A* = {*a*_01_, *a*_02_,…, *a*_*NN*_}, where each *a*_*ij*_ represents the transition probability from state *i* to state *j* and ∑_*j*=1_^*N*^ *a*_*ij*_ = 1  for  all  *i*;
(iii) a set of observation likelihoods (emission probabilities) *B* = {*b*_*j*_(**o**_*t*_)}, where each term represents the probability of an acoustic observation vector **o**_*t*_ being generated from a state *j*. In practice, *b*_*j*_(**o**_*t*_) is modelled as a weighted sum of Gaussian probability density functions (a Gaussian mixture):
  (4) $$\begin{array}{c} b_{j}\left(o_{t}\right)=\sum_{k=1}^{K}C_{jk}N\left(o_{t},\mu_{jk},\Sigma_{jk}\right), \end{array}$$

where *K* denotes the number of mixture components, *C*_*jk*_ is the weight for the *k*-th mixture component satisfying ∑_*k*=1_^*K*^ *C*_*jk*_ = 1, and *N*(**o**_*t*_, ***μ***_*jk*_, Σ_*jk*_) denotes a single Gaussian density function with mean vector ***μ***_*jk*_ and covariance matrix Σ_*jk*_ for state *j*. 

The topology of an HMM reflects the structure of the process that is modelled, and the left-to-right topology presented in Figure 7 is commonly used to model subword units (phonemes) which can be concatenated to form words [30]. This topology was used for all the phoneme HMMs of the baseline ASR system, and 10 Gaussian mixture components were considered for each state.

The training speech corpus (set of acoustic observations) was coded into MFCC feature vectors with the HTK module *HCopy*. The front end used 12 MFCCs plus energy, delta, and acceleration coefficients [30].

Then, the supervised training of the HMMs with the speech corpus (labelled at the phonetic level) was performed with the Baum-Welch and Viterbi algorithms by using the following HTK modules [30].

- *HInit* was used for the individual initialization of the phoneme HMMs. In this case, the initial HMM parameters are estimated by iteratively computing Viterbi alignments between the coded training speech corpus and the associated phonetic labels.
- *HRest* was used to refine the initialized parameters obtained with *HInit*. Re-estimation of the parameters of the individual HMMs is performed with the Baum-Welch algorithm and the labelled training corpus.
- *HERest* was used to further refine the HMMs initialized with *HInit/HRest* with a process called *embedded training*. In contrast to individual HMM training as performed by *HInit/HRest*, embedded training consists in re-estimating the parameters of all HMMs in parallel with the Baum-Welch algorithm.

#### 4.1.2. Lexicon

The lexicon, or phonetic dictionary, was made at the same time as the phonetic labelling of the speech corpus. The phoneme sequences that formed each word in the vocabulary were defined by spectral and perceptual analysis as commented in Sections 2 and 3.

#### 4.1.3. Language Model *L*(Pr(*W*))

Word-bigram language models (2-grams) were estimated from the word transcriptions of the corpus. This was suitable given the size of the training corpus (see Table 3). The following HTK modules were used for this purpose.

- *HLStats* was used to compute label statistics for the purpose of generating a language model. These statistics are estimated from the word transcriptions of the speech corpus and consist in, for example, the probabilities of occurrence of each single word in the corpus (unigram probabilities; see Table 3). If configured to estimate bigram probabilities, it provides the associated probabilities of occurrence for the different pairs of words found in the word transcriptions. For unseen pairs of words, *backed-off* bigram probabilities can be estimated from the unigram probabilities.
- *HBuild* was used to build a word network with the statistics estimated with *HLStats*. This module generated the statistical language model for the baseline ASR system.

An important parameter to control the influence of the language model in the recognition process is the *scale grammar factor*. This factor is defined as the amount by which the language model probability is scaled before being added to each token as it transits from the end of one word to the start of the next [30]. As this factor increases, the recognizer relies more on the language model instead of the acoustic signal to predict what the speaker said (e.g., the language model restrictions have more importance). The module *HVite* (see Section 4.1.4) allows the adjustment of the scale grammar factor during the recognition process, and for this work a value of 10 was used.

#### 4.1.4. Search Algorithm

Speech recognition was performed with the Viterbi algorithm implemented with the module *HVite* of HTK [30]. This module takes as input the coded speech to be recognized and integrates the elements described in Sections 4.1.1, 4.1.2, and 4.1.3 for the estimation of $\hat{W}$ (see (3)). Internally, the features of the input speech are compared with the learned patterns of the phoneme HMMs. The sequence of HMMs that describe the speech signal with a maximum likelihood is restricted to form valid words with the integration of the lexicon. Then these words are restricted with the information of the language model to form valid sequences or sentences which are the main output of the recognition process ($\hat{W}$).

#### 4.1.5. Speaker Adaptation

Note that the Mixtec ASR baseline system only will show good performance when tested by the native speaker used to build the training speech corpus. For its use by non-native speakers (the target users) this is a disadvantage. Non-native speakers were considered the target users because it is important to arouse the interest in the language of the population with more presence in Mexico, the Spanish-speaking people. This may contribute to a change in attitude towards not only the language, but also towards the culture itself, which can be more beneficial for the purposes of preservation.

Commercial ASR systems are trained with thousands or millions of speech samples from different speakers, which leads to speaker-independent (SI) systems. When a new user wants to use such system, it is common to ask the user to read some words or narratives (adaptation stimuli) to provide speech samples that will be used by the system to adapt the SI acoustic models to the patterns of the user's voice. SI ASR systems are robust enough to get benefits by the implementation of adaptation techniques such as MAP or MLLR [28, 30].

For this work there are challenges given by the wide range of variations in tones and pronunciations and the limited availability of native speakers to obtain training speech corpora. Because of this situation, the development of a Mixtec SI ASR system is not achievable. Thus, the use of a speaker adaptation technique on this one-native speaker ASR system was studied.

**Figure 8:**
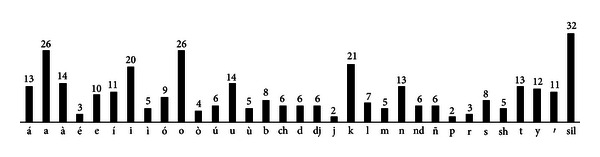
Frequency distribution of phonemes in the Mixtec adaptation stimuli.

Maximum likelihood linear regression (MLLR) [30, 31] was the adaptation technique used for the native ASR baseline system in order to make it usable for non-native speakers. A selection of words from the Mixtec speech corpus was defined to allow the user to provide enough speech samples from the phonemes listed in Table 2 and Figure 5. These words are shown in Table 4 and have the frequency distribution of phonemes shown in Figure 8, which has a correlation coefficient of 0.6543 with the distribution of the speech corpus (Figure 5). Hence it was considered that the adaptation samples were representative of the speech corpus.

MLLR is based on the assumption that a set of linear transformations can be used to reduce the mismatch between an initial acoustic model set and the adaptation data. In this case, these transformations were applied to the mean and variance parameters of the Gaussian mixtures of the Mixtec baseline HMMs, being performed in two steps with the module *HERest* of the HTK software.

(i)  Global adaptation: a global base class was used to specify the set of HMM components that share the same transformation. A first execution of *HERest* was performed to generate a global transformation that was applied to every Gaussian component of the baseline HMMs.

(ii)  Dynamic adaptation: in the second execution of *HERest*, the global transformation was used as an input transformation to adapt the model set, producing better frame/state alignments which were then used to estimate a set of more specific transformations by using a regression class tree. For this work, the regression class tree had 32 terminal nodes [30] and was constructed with the module *HHEd*. The regression class tree is important to cluster together components that are close in acoustic space, so they can be transformed in similar way. Thus, the transformations obtained with the second execution of *HERest* were more specific to certain groupings of Gaussian components and were estimated according to the “amount” and “type” of adaptation data that was available (see Table 4). Because each Gaussian component of an HMM belongs to one particular base class, the tying of each transformation across a number of mixture components can be used to adapt distributions for which there were no observations at all. With this process all models can be adapted, and the adaptation process is dynamically refined when more adaptation data becomes available [30].

### 4.2. Mixtec-to-Spanish/Spanish-to-Mixtec Speech Translators

**Figure 9:**
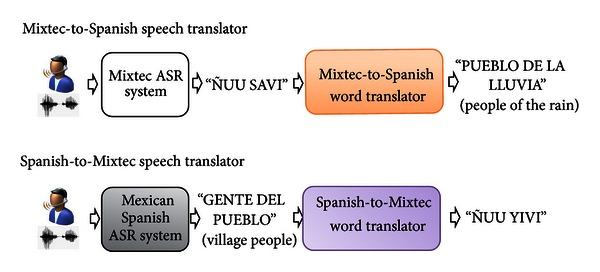
Integration of the baseline Mixtec ASR for other speech applications.

The baseline Mixtec ASR was integrated into two translation systems as shown in Figure 9. The baseline Mexican Spanish ASR system was also built with a native speaker, following the phonetic definitions of the Master in Hispanic Linguistics Javier Octavio Cuétara [25] (see Mexbet in Table 2). The details of this system, with the same elements as the Mixtec ASR system, are freely available in [32] and thus will not be reviewed in this paper. Instead, the details of the translation systems will be explained.

Speech translation is a difficult area of research as there are systematic, idiosyncratic, and lexical differences (translation divergences) between source and target languages [28]. Hence, direct translation (word by word) can lead to unsatisfactory results. For this work, the approach of statistical machine translation (MT) with weighted finite state transducers (WFSTs) was used [28, 33, 34]. In the Statistical MT, translation is performed from a source language *S* to a target language *T* where the best translated sentence into the target language $\hat{T}$ is the one whose probability Pr(*T* | *S*) is the highest. Based on the noisy channel model and the Bayes' rule [28], $\hat{T}$ can be estimated as

(5) T^=arg max⁡T  Pr(T ∣ S)=arg max⁡TPr⁡(S ∣ T)Pr(T)Pr(S)=arg max⁡T  Pr(S ∣ T)Pr(T).

From (5), Pr(*S* | *T*) is termed the *translation model* and Pr(*T*) the *language model* of the target language. For the Mixtec (*X*) to Spanish (*P*) translator *S* → *X* and *T* → *P*, and (5) becomes

(6) P^=arg max⁡P  Pr(P ∣ X)=arg max⁡PPr(X ∣ P)Pr(P)Pr(X)=arg max⁡P Pr⁡(X ∣ P)Pr(P).

The following transducers were defined to compute the elements of (6):

- *X*, the sequence of words of the incoming sentence in Mixtec (for the Spanish-to-Mixtec translator, the incoming sequence is *P*);
- TM_*XP*_, the translation model, which maps the sequences of words from *X* to the most representative words in *P*, which depends on Pr(*X* | *P*) (for the Spanish-to-Mixtec translator, the model is TM_*PX*_);
- *P*, the language model Pr(*P*), which is estimated from the word-bigram language model of the Spanish translations of the Mixtec narratives. For the Spanish-to-Mixtec translator, the language model Pr(*X*) consists of the one estimated from the Mixtec speech corpus as presented in Section 4.1.

Thus, the Mixtec-to-Spanish process of estimating the most probable sequence of words $\hat{P}$ given *X* can be expressed as

(7) $$\begin{array}{c} \hat{P}=\tau^{*}\left(X\circ {\text{TM}}_{XP}\circ P\right). \end{array}$$

In contrast, the Spanish-to-Mixtec process of estimating $\hat{X}$ given *P* can be expressed as

(8) $$\begin{array}{c} \hat{X}=\tau^{*}\left(P\circ {\text{TM}}_{PX}\circ X\right). \end{array}$$

In (7) and (8), τ* denotes the operation of finding the most likely path through a transducer and ∘ denotes composition of transducers [34]. It is important to observe that the translation systems were restricted by the stored vocabulary of the Mixtec speech corpus. Thus, the language model of the baseline Spanish Mexican ASR system consisted of the Spanish translation of the Mixtec narratives. The translation from Mixtec to Spanish was assisted by Professor Maximino for the modelling of Pr(*X* | *P*) and Pr(*P* | *X*). The probabilities of the translation models were estimated by counting single and multiple word alignments between Mixtec and Spanish words following the *phrase alignment* approach described in [28]. The probabilities of the word bigram language models were also estimated by counting.

**Figure 10:**
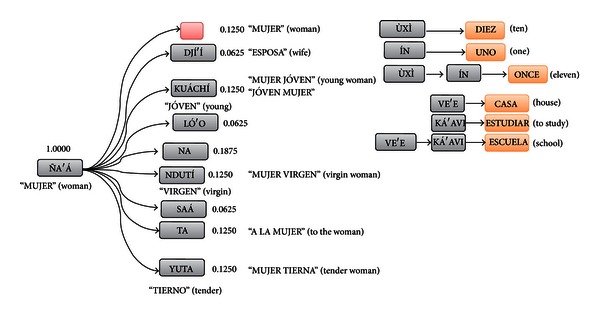
Example of the Mixtec-to-Spanish translation model (TM_*XP*_).

**Figure 11:**
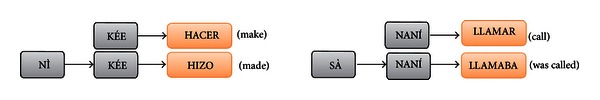
Example of verb tenses in the Mixtec-to-Spanish translation model (TM_*XP*_).

**Figure 12:**
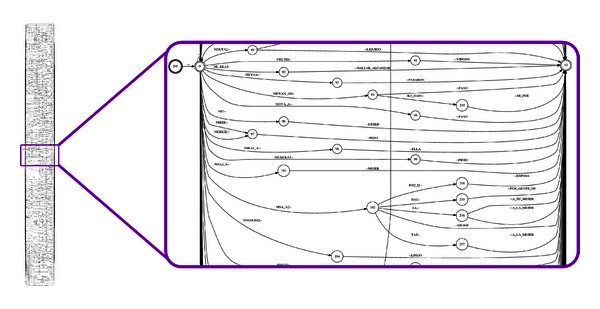
Fragment of the TM_*XP*_ transducer.

In Figures 10 and 11 some graphic examples of the translation model are shown. In Figure 10, “ña'á” means “woman” and if followed by “djí'í” (with a probability of 0.0625) then means “wife”. In this case, one (“ña'á”) and two (“ña'á djí'í”) words in the source language are aligned to a single word in the target language (“woman”, “wife”). Thus, some words combine their meanings to form concepts that relates to them. For example, “ùxì” means “ten”; however if it is followed by “ín”, which means “one”, then the meaning becomes “eleven”. The same applies to “school” which is translated from “ve'e” (“house”) and “ká'avi” (“to study”) (e.g., house to study). Something similar happens to verb tenses as presented in Figure 11. A fragment of the real translation model transducer for the Mixtec-to-Spanish translator is shown in Figure 12 (for visibility purposes the probabilities were removed).

The definitions of the transducers for the Spanish-to-Mixtec translator were performed following the same methodology for the Mixtec-to-Spanish translator. Thus, they will not be discussed in this section. Finally, the implementation tool for the transducers was the FSM Library [34, 35] from AT&T, and for computational convenience, all probabilities were converted into logarithmic probabilities.

### 4.3. Graphical User Interfaces

**Figure 13:**
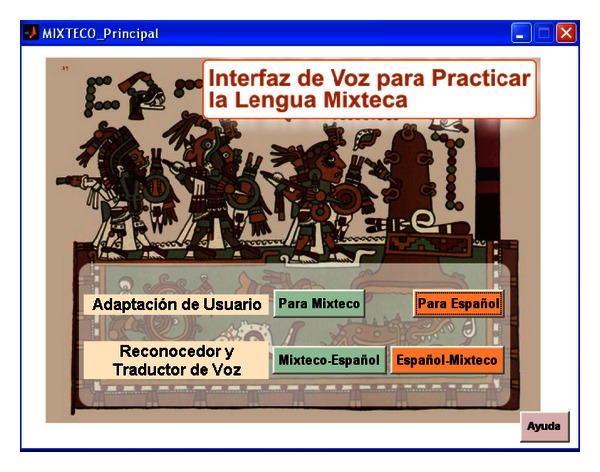
Main window of the speech interface with the Mixtec ASR system.

In Figure 13 the main window of the speech interface for the applications of the Mixtec and Spanish ASR systems is shown. The programming platform was MATLAB 2008 using the GUIDE toolbox. There are two main panels: “Adaptación de Usuario” (Speaker Adaptation) and “Reconocedor y Traductor de Voz” (Speech Recognizer and Translator).

**Figure 14:**
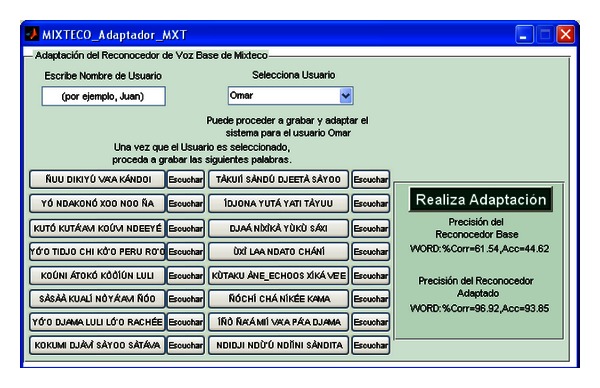
Speaker adaptation module for the baseline Mixtec ASR system.

**Figure 15:**
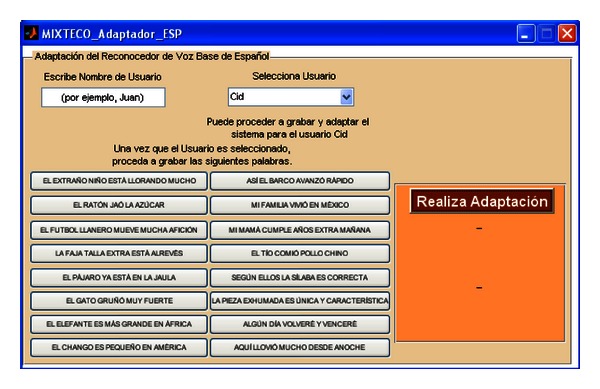
Speaker adaptation module for the baseline Spanish ASR system.

The first panel has two buttons, where each one leads to a module to perform adaptation of the associated baseline ASR system for its use by a new non-native speaker. Thus, the button “Para Mixteco” (For Mixtec) opens the window shown in Figure 14, and the button “Para Español” (For Spanish) opens the window shown in Figure 15.

The adaptation modules work as follows: in both Figures 14 and 15, there are two fields, “Escribe Nombre de Usuario”, (Write Name of the User) and “Selecciona Usuario” (Select User), which are set to register a new user to start the adaptation process. When the user writes his/her name in the field “Escribe Nombre de Usuario” the interface updates the list of the pop-up menu “Selecciona Usuario” and creates the directories to store the MLLR transformations. If the user is already registered, an informative message is shown. Once this task is finished the user can proceed to record the adaptation data, which is shown on each of the buttons located under these fields. For both Mixtec and Spanish, the stimuli consist of 16 sentences. Note that in Figure 14 the adaptation sentences defined in Table 4 are shown. 

As the interfaces are considered for non-native speakers of the Mixtec language, the stimuli buttons of Figure 14 are accompanied by a button labelled “Escuchar” (Listen). The user by pressing that button can listen to the native pronunciation of the associated stimuli sentence as support to practice the pronunciation before recording the adaptation speech. The speech is recorded, by pressing the stimuli button, where one click starts the recording and another finishes it. After all sentences are recorded the user just needs to press “Realiza Adaptación” (Perform Adaptation) to perform MLLR adaptation of the HMMs of the baseline ASR. The adapted models and transformations are stored (or updated) into the personal directories of the user. After the adaptation process some information about the performance of the (now) speaker-adaptive system is shown. Under “Precisión del Reconocedor Base” (Accuracy of the Baseline Recognizer) the performance of the unadapted baseline is shown, while the performance of the adapted baseline is shown under “Precisión del Reconocedor Adaptado” (Accuracy of the Adapted Recognizer). The metric of performance for the ASR systems is the percentage of word recognition accuracy, Acc, which is computed as

(9) $$\begin{array}{c} \text{Acc}=\frac{N-D-S-I}{N}\times 100. \end{array}$$

In (9), *D*, *S*, and *I* are deletion, substitution, and insertion errors in the recognized speech (text output of the ASR system). *N* is the number of words in the correct ASR's output [30]. As presented in Figure 14, the unadapted ASR system achieved an accuracy of 44.62% on the adaptation stimuli, and 93.85% after the system was adapted with the spoken stimuli for the non-native speaker Omar.

**Figure 16:**
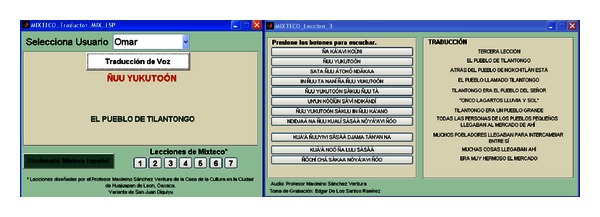
Mixtec-to-Spanish speech translator.

**Figure 17:**
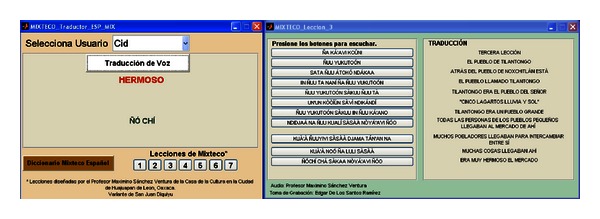
Spanish-to-Mixtec speech translator.

The second panel of the main window has also two buttons, “Mixteco-Español” (Mixtec-Spanish) and “Español-Mixteco” (Spanish-Mixtec), where each one leads to the associated translator, which are shown in Figures 16 and 17. By pressing the “Mixteco-Español” the window shown in Figure 16 is loaded, which enables Mixtec speech recognition, translation to Mexican Spanish, and text-to-speech synthesis. Initially the user must select his/her name in the pop-up menu “Selecciona Usuario” (Select User). When doing this, the MLLR transformations and directories for that user are loaded. Then, by pressing “Traducción de Voz” (Speech Translation) the interface starts to record the speech of the user which has non-native Mixtec pronunciation. When the user finishes, he/she just needs to press again the button “Traducción de Voz”. This starts the recognition process, which displays under the button the recognized Mixtec words (in this case, “ñuu yukutoón”). Then, internally, the interface converts this string (*X*) into a format suitable for its composition with the TM_*XP*_ and *P* transducers and manages the FSM Library to perform this task and provide the most likely translation $\hat{P}$ which is also displayed (in this case, “el pueblo de Tilantongo” = “the town of Tilantongo”). $\hat{P}$ is then given to a speech synthesizer which “reads” these words. For this purpose the Windows XP Speech Application Programming Interface (SAPI) ver 5.0 and the Spanish voice Isabel from ScanSoft were used.

In addition to this function, the user can get access to all the Mixtec narratives by pressing the buttons “1–7” located at the bottom of the window under “Lecciones de Mixteco” (Mixtec Lessons). In Figure 16 the lesson or narrative “3” was selected. There, each button plays a sentence of the narrative which is spoken by the native speaker, and next to them the associated Spanish translation is shown. The same lessons can be accessed from the Spanish-to-Mixtec translator shown in Figure 17. Finally, the user also can access a Mixtec-to-Spanish dictionary by pressing the button “Diccionario Mixteco Español” which lists all Mixtec words in the speech corpus and their equivalents in Spanish.

## 5. Performance of the Speech Applications Built with the Speech Corpus

**Figure 18:**
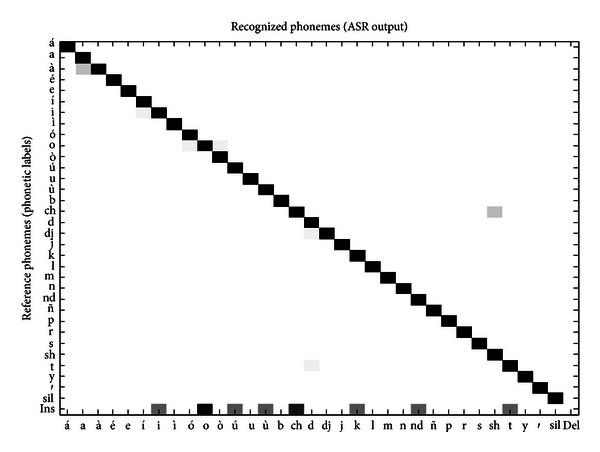
Phoneme confusion matrix of the baseline Mixtec ASR system on the training speech corpus.

Initially the Mixtec ASR system was tested with the training speech corpus to analyze its performance for classification of known data. For the recognition task a scale grammar factor of 10 was used as mentioned in Section 4.1.3. A word recognition accuracy (9) of 97.85% (*N* = 931, *D* = 0, *S* = 10, *I* = 10) was obtained, which is considered high and normal considering that ASR is performed on the training data [30]. This word output was then transcribed at the phonetic level to analyze the performance of phoneme recognition. For this task, the original phonetic labels of the corpus were compared with the phonetic transcription of the word output. In Figure 18 the phoneme confusion matrix obtained from this comparison is presented. Observe that, because no words were deleted in the word output, there are no deleted phonemes in the transcription, and thus, the entries in the column “Del” (Deletions) are zero. In contrast, there are entries in the row “Ins” (Insertions) because in the word output there were inserted words (*I* = 10). Substitution of phonemes (misclassification) is almost non-existent because *S* = 10 and *N* = 931 in the word output. In general, the phoneme recognition accuracy was of 99.10% (*N* = 3655, *D* = 0, *S* = 22, *I* = 11) which is considered important to demonstrate that the modelling of the Mixtec phonemes by the baseline ASR was performed satisfactorily.

Now the tests of the ASR applications on the speech of different users are discussed. Ten non-native speakers, five males (M) and five females (F), were recruited to test the performance of the ASR applications. Prior to using the speech interfaces for adaptation and recognition/translation, all speakers received three hours of informative sessions which were distributed over three days. In these sessions, information about the pronunciation of the Mixtec words from the 7 narratives, including the audios from the native speaker, was reviewed.

**Figure 19:**
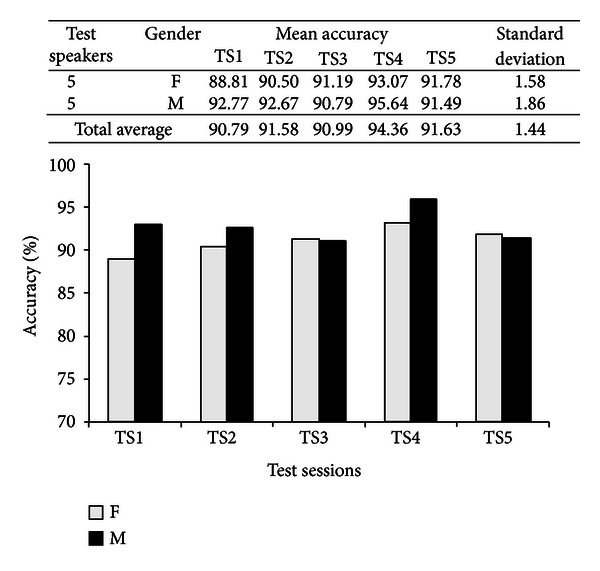
Accuracy of the speaker-adaptive Mixtec ASR system.

**Figure 20:**
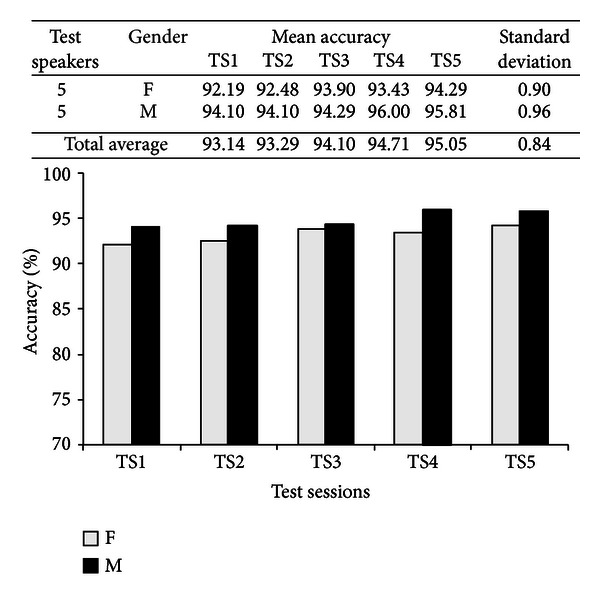
Accuracy of the speaker-adaptive Mexican Spanish ASR system.

After the informative sessions, the speakers used the adaptation interfaces (see Figures 14 and 15) to perform registration and MLLR adaptation. Then, they proceeded to use the recognition/translation interfaces (see Figures 16 and 17). In total, five test sessions were performed, which consisted in the speakers reading three narratives or lessons with different levels of difficulty: 1 (easy level), 3 (medium level), and 6 (hard level). The test narratives in Mixtec were separated into 49 sentences with a total of 202 words, while the narratives in Spanish were separated into 48 sentences with a total of 210 words. The metric of performance of the speech recognizers was the word recognition accuracy (see (9)). The results are presented in Figures 19 and 20 for both recognition systems. 

For the speaker adaptive Mixtec ASR, mean recognition accuracies are within the range of 88.81%–93.07% for the female speakers and within 90.79%–95.64% for the male speakers, achieving a total of 90.79%–94.36%. The mean variability in performance, measured by the standard deviation, across the test sessions is slightly higher for the male speakers than for the female speakers (1.86 > 1.58). On the other hand, for the Mexican Spanish ASR system the mean accuracies are higher, being within the range of 92.19%–94.29% for the female speakers, and 94.10%–96.00% for the male speakers. Consistently, although very slightly, the variability across sessions is higher for the male speakers (0.96 > 0.90). Nevertheless both performances are comparable to human transcription (96%–98%) and commercial ASR for non-native/indigenous speech with vocabularies <1000 words (80%–96%) [36].

Note that performance variability is significantly higher for the Mixtec system (1.44 > 0.84), which may be due to the system being used by non-native speakers (who are natives of the other system). However the performance observed in the Mixtec system is very similar across all test sessions to the Spanish Mexican system, and both correlate to each other with a coefficient of 0.682.

For a metric to measure the quality of a translation, there are many techniques as the translation word error rate (TWER) and the character error rate (CER) [33]. For this work, TWER was used for the assessment of the translations. TWER is defined as the minimum number of word substitution, deletion, and insertion operations required to convert the target sentence provided by the translation system into the reference translation, divided by the number of words of the reference translation. For the Mixtec-to-Spanish and Spanish-to-Mixtec translators, the reference translations are shown next to each lesson (see Figures 16 and 17). Hence, while identifying the deletion, substitution, and deletion errors in the recognition tasks (to measure the accuracy), the same kind of errors was identified to assess the TWER. In Tables 5 and 6 the mean TWERs of the Mixtec-to-Spanish and Spanish-to-Mixtec translators are presented. The TWERs are slightly higher for the Mixtec translations, although in general these are less than 20%, which is within the ranges reported by other computer-assisted translation systems [33]. Both results correlate to each other with a coefficient of 0.57379.

The results in Table 5 were compared with those presented in Figure 19. It was assumed that high recognition accuracy was needed to get good translation levels (low TWERs), although this is also dependent on the translation model. The results in Table 5 correlate to the recognition accuracies presented in Figure 19 with a coefficient of −0.62237, indicating a significant inverse relationship. The same was obtained when the results in Table 6 were compared with those in Figure 20, obtaining a correlation coefficient of −0.72027. Thus there is a significant relationship between low TWER and high ASR accuracy.

## 6. Conclusions and Future Work

In this paper the development of a Mixtec speech corpus and two speech applications (built with this resource) was presented. The development of speech corpora is very challenging for the Mixtec language given the high diversity of tones, alphabets, and vocabulary, which vary among regions. Also because of the almost non-existent formal knowledge of the native speakers about the grammar, syntax, and writing rules of their Mixtec language variation. Hence, although there are many people who speak the language, they do not know how to write it or read it, and this restricts greatly the development of speech corpora and also other means to preserve the language.

Hence, only native professional linguists could be suitable to develop the resource. However the availability of people with this background, or with formal knowledge, is not broad, which makes it very difficult to develop large speech corpora. The problem had to be delimited, and thus, it was considered to focus on a single variant and one native speaker with knowledge to ensure accurate phonetic and orthographic labelling of native speech samples for purposes of developing speech applications.

The steps followed to develop the single-speaker corpus were presented in Sections 2 and 3, and in order to test its usefulness as a resource for the development of ASR systems, two applications were developed: a speaker-adaptive Mixtec ASR system and a Mixtec-to-Spanish/Spanish-to-Mixtec translator. These were presented in Section 4, and the results presented in Section 5 give confidence about the attainable use of the corpus for other applications as language learning interfaces.

With recognition accuracies up to 94% across different test sessions with ten non-native speakers, the developed applications can be used for basic learning activities. For formal language learning tasks, the approach must be different in order to assess the pronunciation of a non-native speaker, and it would be essential to have native speech data from female speakers. On the other hand, the translation between Spanish and Mixtec showed TWERs around 20%, which are within ranges of well-documented computer-assisted translation performance. The translation field is as difficult as the recognition field, and for the Mixtec language much more work is needed to develop robust performance for larger vocabularies and different variations. However, the advances presented in this paper can be used as starting point for future researchers in Mixtec or other under resourced languages.

Among the ongoing and future work, the following can be mentioned:

- to develop techniques to increase the performance of the native speaker-adaptive (SA) ASR system (when acoustic resources are limited for supervised training);
- to increase the training speech corpus: add more vocabulary words and increase the complexity to the narratives, recruit more native speakers (both genders) in order to develop a native SI ASR system, and test the system with more users with different levels of expertise in the Mixtec language (preferably native speakers);
- to improve the GUI to increase usability: incorporate learning methodologies to extend the use of the ASR system for users that do not have previous knowledge of the language (with no informative sessions) and integrate a measure of performance for the level of knowledge or practicing that the user gets by using the speech application;
- to extend the modelling of the grammar rules of the translation and language models to reduce TWER in the translation systems;
- to develop a TTS synthesizer for the Mixtec variant of San Juan Diquiyú.

## Figures and Tables

**Table 1:** Examples of Mixtec words with tones.

Word	Meaning
ñoó	Night
ñoo	Town
ño o	Palm
yukú	Who
yuku	Mountain
yuku	Leaf

**Table 2:** Repertoire of Mixtec phonemes.

Description	IPA	Mixtec	Mexbet
Voiceless bilabial stop	p	p	p
Voiceless dental stop	t	t	t
Voiceless velar stop	k	k	k
Voiced bilabial stop	b	b	b
Voiced dental stop	d	d	d
Voiced velar stop	g	g	g
Voiceless palatal affricate	$\overset{⌢}{ʧ}$	ch	tS
Voiceless palatoalveolar fricative	*∫*	sh	
Voiceless labiodental fricative	f		f
Voiceless alveolar sibilant	s	s, dj	s
Voiceless velar fricative	x	j	x
Voiced palatal fricative	*Ӡ*	y	Z
Bilabial nasal	m	m	m
Palatal nasal	*ɲ*	ñ	ñ
Alveolar nasal	n	n	n
Alveolar lateral	l	l	l
Alveolar trill	r	r	r
Alveolar flap	*ɾ*		r(
Close front unrounded vowel	i	i, í, ì	i
Close-mid front unrounded vowel	e	e, é	e
Open front unrounded vowel	a	a, á, à	a
Close-mid back rounded vowel	o	o, ó, ò	o
Close back rounded vowel	u	u, ú, ù	u
Glottal stop	*ʔ*	'	
Additional		nd, ng	ks
			_D, _G
			_N, _R
		sil	sil

**Table 3:** Frequency of the vocabulary in the Mixtec speech corpus.

No.	Word	Freq.
1	ÀNE′ECHOOS	1
2	ÁN	2
3	ÁTOKÓ	2
4	CHÁ	8
5	CHÁNÍ	1
6	CHÁNÍTÀ	1
7	CHI	11
8	DIKIYÚ	2
9	DJÀVÌ	2
10	DJAÁ	5
11	DJAMA	6
12	DJE′E	2
13	DJEETÀ	1
14	DJÍ′Í	2
15	DJÍO	4
16	DJIÁ	2
17	I	2
18	Í′A	1
19	ÍDJONA	1
20	ÍN	10
21	ÍNI	5
22	ÍÑÒ	2
23	IIN	4
24	INÍXOO	7
25	KÀKÀ	2
26	KÁ′A	2
27	KÁ′ANO	4
28	KÁ′AVI	26
29	KÁN′AN	4
30	KÁNDOI	1
31	KAMA	2
32	KÌVÌ	4

		129

33	KIÁ	2
34	KIDJÍ	1
35	KO′OAN	2
36	KÒ′O	4
37	KÒÒÍÚN	17
38	KOÍÑO	1
39	KOKUMI	2
40	KOÑO	1
41	KOÓ	4
42	KOTO	1
43	KOÚN′UN	5
44	KOÚNI	2
45	KOÚSA	2
46	KOÚVI	2
47	K*Ù*ÌÀ	3
48	K*Ù*TAKU	1
49	KUÀ′À	19
50	KUÀ′ÀKÁ	1
51	KUÁ′A	2
52	KUÁCHÍ	5
53	KUALÍ	3
54	KUI′Í	3
55	KUÍ	4
56	KUÍI	2
57	KUKU	2
58	KUTÁ′AVI	6
59	KUTÓ	1
60	KUÚ	2
61	LAA	5
62	LÓ′O	5
63	LULI	2
64	ME′Í	1

		113

65	MIÍ	7
66	NA	32
67	NÀ	10
68	NÀVE′E	2
69	NÁ	4
70	NÁ′ANO	2
71	NANÍ	3
72	NAVA′ATI	2
73	NDÁ′A	4
74	NDA′Á	4
75	NDÁ′AITA	1
76	NDÁKAA	2
77	NDÁNDEI	1
78	NDAKÁNI	1
79	NDAKONÓ	2
80	NDATO	5
81	NDEEYÉ	2
82	NDÌÍNI	2
83	NDÌKUA	2
84	NDÌTIVI	1
85	NDÍ	5
86	NDICHÍ	4
87	NDIDJAÁ	4
88	NDIDJI	4
89	NDIKÁNDÍ	17
90	NDISÁN′AN	1
91	NDOVA′A	3
92	ND*Ù*′Ú	1
93	NDUTÁ	3
94	NDUTATÍ	2
95	NDUTÍ	5
96	NE′EKÁ	1

		139

97	NÌXÌKÀ	1
98	NÌYA′A	2
99	NÌYAN′AN	1
100	NÍ	9
101	NÍKÉE	4
102	NIKÀ′A	1
103	ÑA	34
104	ÑA′Á	22
105	ÑÀ	2
106	ÑÓCHÍ	4
107	ÑÓO	8
108	ÑUU	43
109	ÑUUYIVI	4
110	NÒO	4
111	NÒYÁ′AVI	4
112	NOO	2
113	NOÓ	3
114	OON	2
115	PÁ′A	3
116	PERU	3
117	RACHÉE	3
118	RO′O	3
119	SA′A	2
120	SÀ′À	5
121	SÀDJANÁNI	1
122	SÀDJANDÁKU	7
123	SÀKAA	2
124	SÀKEE	2
125	SÀKONI	8
126	SÀKOÓ	2
127	SÀKOON	2
128	SÀKUÁ	2

		195

129	SÀKUU	17
130	SÀNANÍ	3
131	SÀNDAKÀÀ	5
132	SÀNDITA	7
133	SÀNDÚ	1
134	SÀNDUKÚ	2
135	SÀSA′A	2
136	SÀSÀÀ	6
137	SÀTÁVA	1
138	SÀTAÁ	2
139	SÀVÌ	17
140	SÀXI′Í	2
141	SÀXITO	2
142	SÀYOO	11
143	SÁXI	1
144	SAÁ	2
145	SANDOI	3
146	SATA	2
147	TA	19
148	TA′Á	2
149	TÀ	40
150	TÀKUIÍ	7
151	TÀKUIÍAN	1
152	TÀYUU	2
153	TÁ	26
154	TÁN′AN	4
155	TÁNDÀ′À	9
156	TÁTÁ	5
157	TAA	2
158	TAAN	1
159	TATA	2
160	TI′A	1

		207

161	TIDJO	4
162	TÓO	2
163	TÓOKA	1
164	TOÓN	1
165	TÚKU	2
166	*Ù*XÌ	7
167	UN′UN	17
168	VA′A	18
169	VE′E	4
170	VE′ECHÓON	1
171	VIKO	5
172	XÌ′Ì	1
173	XÍ′Í	8
174	XÍKÁ	1
175	XÍTI	1
176	XOO	2
177	YÁVI	3
178	YATI	2
179	YIÍ	2
180	YITO	8
181	YÒO	1
182	YÓ	12
183	YÓ′O	5
184	YOO	1
185	YOÓ	4
186	YU′Ú	2
187	Y*Ù*′*Ù*	2
188	Y*Ù*K*Ù*	1
189	YÚKU	1
190	YUKUTOÓN	25
191	YUTA	2
192	YUTÁ	2

		148

**Table 4:** Adaptation stimuli words.

ÑUU DIKIYÚ VA′A KÁNDOI
YÓ NDAKONÓ XOO NOO ÑA
KUTÓ KUTÁ′AVI KOÚVI NDEEYÉ
YÓ′O TIDJO CHI KÒ′O PERU RO′O
KOÚNI ÁTOKÓ KÒÒÍÚN LULI
SÀSÀÀ KUALÍ NÒYÁ′AVI ÑÓO
YÓ′O DJAMA LULI LÓ′O RACHÉE
KOKUMI DJÀVÌ SÀYOO SÀTÁVA
TÀKUIÍ SÀNDÚ DJEETÀ SÀYOO
ÍDJONA YUTÁ YATI TÀYUU
DJAÁ NÌXÌKÀ Y*Ù*K*Ù* SÁXI
*Ù*XÌ LAA NDATO CHÁNÍ
K*Ù*TAKU ÀNE′ECHOOS XÍKÁ VE′E
ÑÓCHÍ CHÁ NÍKÉE KAMA
ÍÑÒ ÑA′Á MIÍ VA′A PÁ′A DJAMA
NDIDJI ND*Ù*′Ú NDÌÍNI SÀNDITA

**Table 5:** TWER of the Mixtec-to-Spanish translator.

Source: Mixtec		Target: Mexican Spanish					
Test speakers	Gender		Mean TWER				Standard deviation
		TS1	TS2	TS3	TS4	TS5
5	F	19.90	18.86	18.67	18.38	19.33	0.60
5	M	18.29	18.95	17.81	17.05	17.24	0.78

Total average		19.10	18.90	18.24	17.71	18.29	0.56

**Table 6:** TWER of the Spanish-to-Mixtec translator.

Source: Mexican Spanish		Target: Mixtec					
Test speakers	Gender		Mean TWER				Standard deviation
		TS1	TS2	TS3	TS4	TS5
5	F	20.59	19.80	18.61	20.40	19.01	0.86
5	M	18.81	19.50	18.91	18.22	19.31	0.50

Total average		19.70	19.65	18.76	19.31	19.16	0.39
